# Leucocytoclastic Vasculitis, Cryoglobulinemia, or Plasma Cell Leukemia: A Diagnostic Conundrum

**DOI:** 10.7759/cureus.16832

**Published:** 2021-08-02

**Authors:** Hycienth Ahaneku, Ruby Gupta, Nwabundo Anusim, Chukwuemeka A Umeh, Joseph Anderson, Ishmael Jaiyesimi

**Affiliations:** 1 Hematology and Oncology, Beaumont Health, Royal Oak, USA; 2 Internal Medicine, Beaumont Health, Hemet, USA; 3 Hematology and Oncology, Beaumont Health, Royal Oak, USA

**Keywords:** plasma cell leukemia, cryoglobulinemia, multiple myeloma, bortezomib, lenalidomide, autologous stem cell transplant

## Abstract

Plasma cell leukemia is rare and could be life-threatening. Even rarer and equally life-threatening is cryoglobulinemia. Both of them occurring together paints a grim clinical picture. We present the case of a 63-year-old male with plasma cell leukemia complicated by cryoglobulinemia with skin lesions. The report briefly reviews the clinical and diagnostic characteristics of plasma cell leukemia and well as available treatment options. It also highlights the need to consider non-chemotherapy-based regimens and clinical trials in the care of plasma cell leukemia patients.

## Introduction

Plasma cell dyscrasias are a group of hematologic malignancies characterized by clonal proliferation of plasma cells. They cover a spectrum ranging from the premalignant monoclonal gammopathy of undetermined significance (MGUS) to the more aggressive multiple myeloma (MM) and plasma cell leukemia (PCL). PCL is often seen as the most aggressive plasma cell neoplasm. PCL accounts for less than 4% of plasma cell neoplasms and its population incidence is about one in a million, making it one of the rarest of plasma cell neoplasms [1].

About 60% of PCL occur de novo as primary PCL while the rest occur secondarily from MM [2]. Generally, PCL carries poorer survival outcomes compared to MM [3,4]. Due to its rarity, most of what we know about PCL derives from retrospective studies [1]. In a review of plasma cell leukemia, most patients were male, African Americans. Like patients with MM, most PCL present with fatigue from anemia, renal failure, and bone pains from lytic lesions [5].

While most cases of PCL present with features highlighted above, few cases have reported cryoglobulinemia-induced skin necrosis [6]. We present the case of a 63-year-old male who presented with lower extremity skin necrosis and was later diagnosed with cryoglobulinemia and PCL.

## Case presentation

A 63-year-old African American male presented to the hospital with a painful blistering rash over his lower extremities. The patient’s problem started 10 days prior with acute pain in the toes bilaterally, which quickly progressed to black discoloration of the skin with patchy loss of sensation in both feet. Twenty-four to 48 hours later, the lesions progressed to involve the skin of both legs, thighs, and arms in a varied patchy fashion. A review of the system revealed fatigue and 10-pound weight loss in the preceding month.

**Figure 1:**
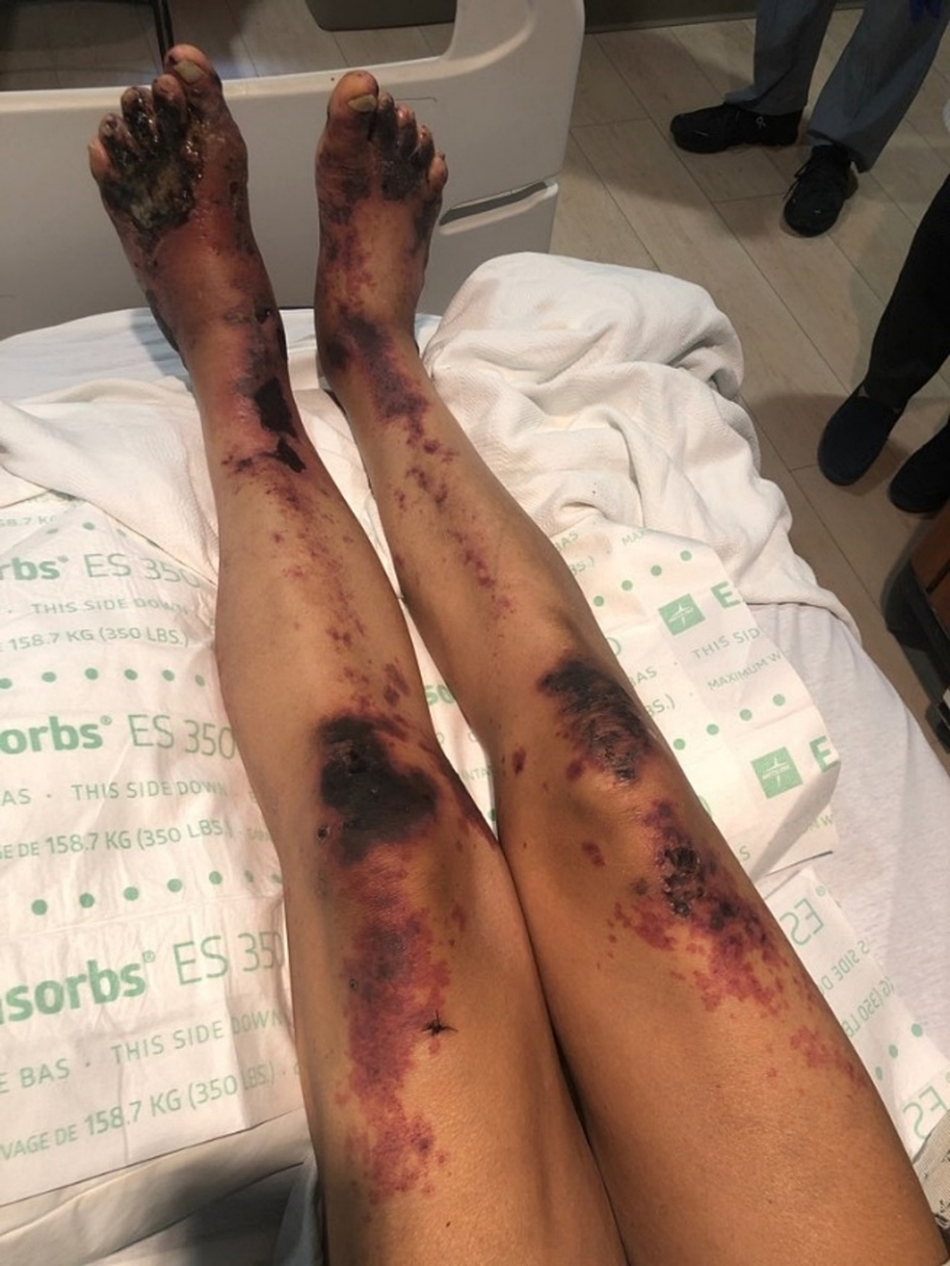
Necrotic vasculitic lesions in both lower limbs

Examination revealed black patchy areas of ulcers, necrosis, and discoloration in the feet, legs, thighs, upper arms, and ears (Figure 1).

There were patchy areas of tenderness and reduced sensation around the discolored areas. Results of initial workup were as follows: white blood count (WBC) 19,800/cm^3^, hemoglobin (Hb) 9.8, platelets 157,000/cm^3^, sodium (Na) 127 mmol/L, blood urea nitrogen (BUN) 63mg/dL, creatinine 2.85mg/dL, erythrocyte sedimentation rate (ESR) 130mm/hr, c-reactive protein (CRP) 27mg/L, total protein 12.8g/dL, and albumin 2.9g/dL. Peripheral blood smear showed rouleaux formation. Of note, the blood work also revealed plasma cells 2,200/cm^3^.

**Figure 2:**
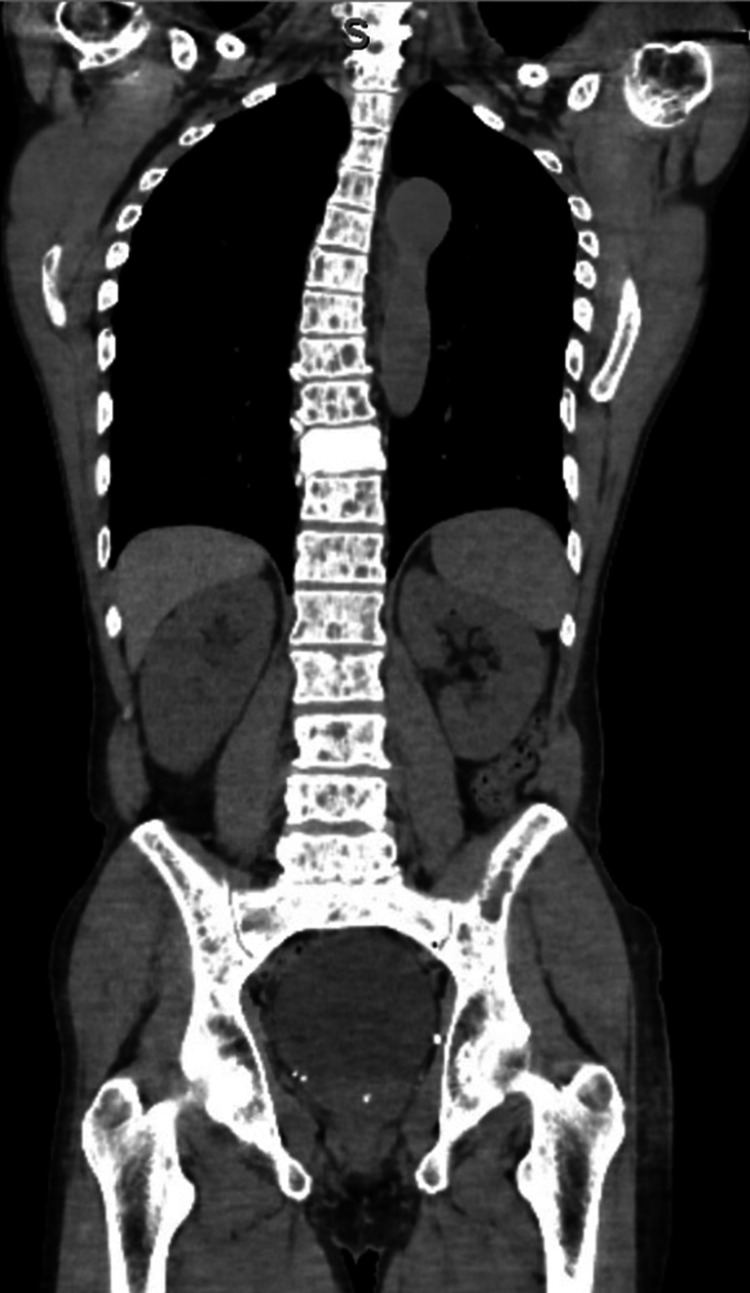
Coronal CT scan showing lytic lesions in the axial skeleton

On account of his clinical picture, there was a concern for malignancy and the patient had a CT chest/abdomen/pelvis showing diffuse abnormal axial skeleton with multiple punched-out, lytic bony lesions (Figure 2).

His constellation of clinical and laboratory features including anemia, renal failure, bony lytic lesions, and a blood smear was suggestive of plasma cell dyscrasia. A serum protein electrophoresis (SPEP) showed a spike in the gamma region with M-protein estimated at 5.4g/dL. Serum immunofixation evaluation (SIFE) showed an IgG lambda monoclonal protein and free lambda protein. IgG was 7,239mg/dL (normal 550-1,650mg/dL), IgM 26 (normal 40-293 mg/dL), and IgA 37mg/dL (normal 70-365mg/dL). Free light chain (FLC) analysis reveals Kappa FLC 0.70mg/dL and free lambda 152.1mg/dL. Beta-2 microglobulin (B2M) was 7.96mg/L. An initial report of bone marrow biopsy showed 90% cellularity, with decreased trilineage hematopoiesis and markedly increased plasma cells (80%) with associated Russel bodies. Flow cytometry on bone marrow specimens was positive for a neoplastic plasma cell population estimated at 72% of total cells analyzed. The aberrant plasma cells expressed CD38 (bright), CD138 (bright), CD45, and lambda monotypic light chain. CD19, CD20, CD56, and CD117 were negative.

For his skin lesions, there was a concern for a vasculitic syndrome. Rheumatologic workup was essentially negative except for low complement C4 and positive cryoglobulin. Initial cryoglobulin level was positive at 81%, a biopsy of skin lesion was less likely leucocytoclastic vasculitis and blood viscosity was 3.1cp. Bilateral lower extremity venous and arterial Doppler ruled out associated deep venous thrombosis (DVT) or peripheral artery disease (PAD) in major arteries.

**Figure 3:**
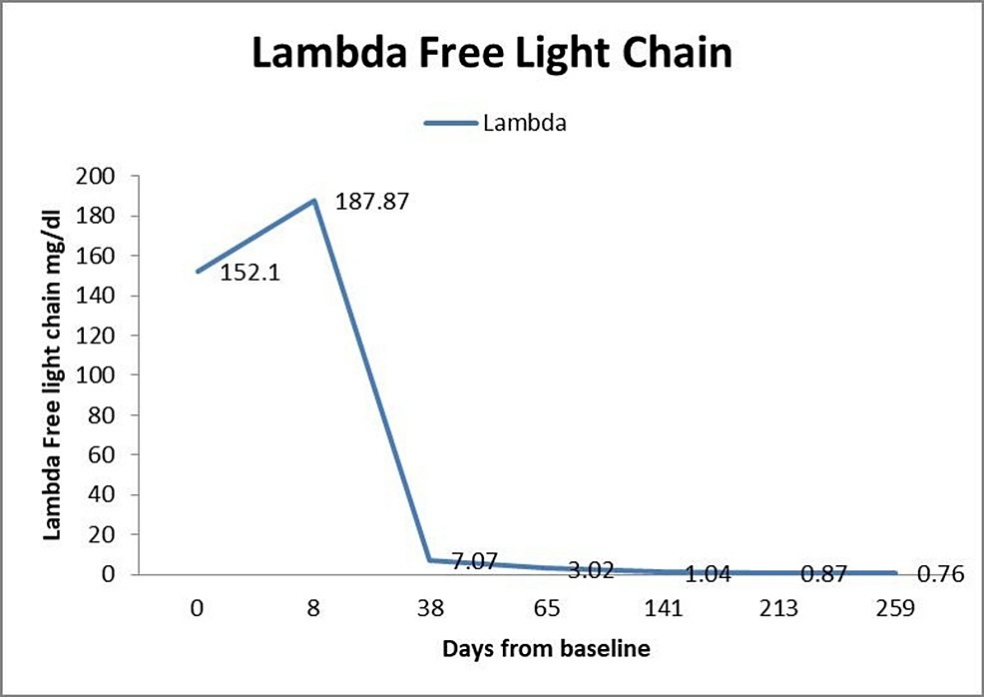
Chart showing a trend in lambda free light chain level over time

**Figure 4:**
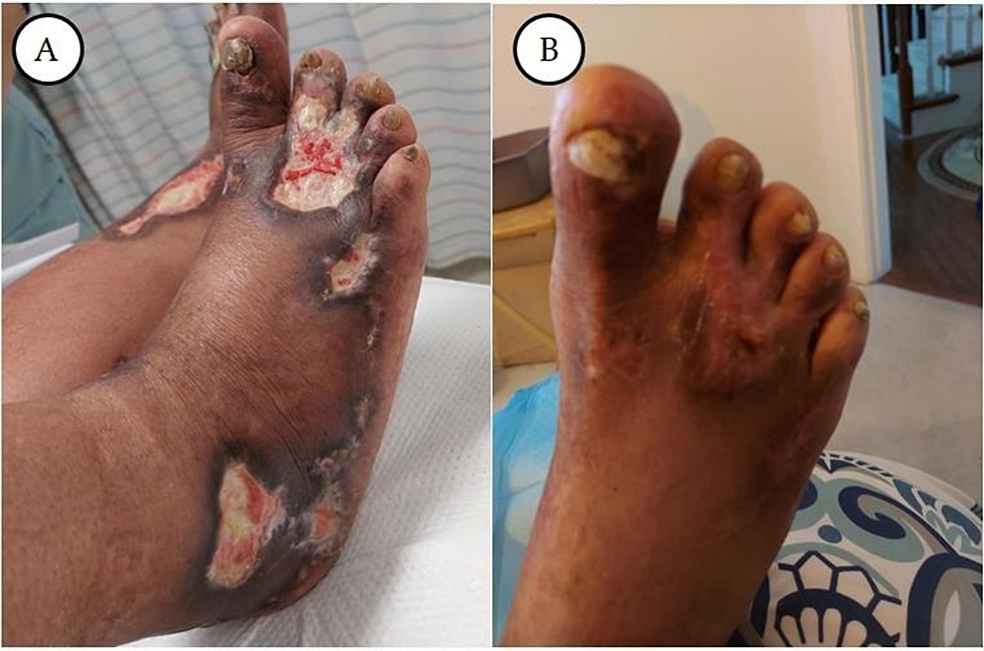
(A) Early stages of necrotic leg ulcers. (B) Significantly healed leg ulcers - six months after.

A working diagnosis of PCL and cryoglobulinemia type 1 was made. On account of his constellation of clinical problems, the patient underwent plasmapheresis. The patient was also started on myeloma-directed treatment, which included bortezomib, dexamethasone, and lenalidomide (started after improved renal function). Over time, the patient's clinical and laboratory features improved. He worked with physical therapy to improve his ambulation. At the time of discharge, cryoglobulin was undetectable, creatinine was 1.63mg/dL (down from 2.85mg/dL at admission), and plasma cell was 400/cm^3^ (down from 2,400cm^3^ at admission). At clinic follow-up about three weeks post-discharge, the patient reported feeling better and skin lesions continued to improve with creatinine being 1.4mg/dL. Bone marrow cytogenetic and FISH studies revealed t(11,14) and IGH/CCND1 gene rearrangements respectively. Repeat SPEP at time of follow up (one month later) showed M protein of 0.4g/dL (down from 5.4g/dL at admission). At the time of manuscript writing, the patient has completed six months of bortezomib, dexamethasone, and lenalidomide with excellent clinical and laboratory response (Figures 3, 4A, 4B). The patient recently underwent stem cell harvesting in preparation for autologous stem cell transplant (ASCT).

## Discussion

Ours is the case of a 63-year-old male who presented with lab findings showing an absolute plasma cell count of 2,200/mL consistent with PCL. PCL stands at the worst extreme of the plasma cell dyscrasias with median survival estimated at seven months [3]. PCL has traditionally been defined as having peripheral plasma counts ≥20% or absolute counts ≥2,000/mL [7]. However, the International Myeloma Working Group suggests that patients with ≥5% peripheral plasma cells or absolute count ≥500/mL should be reassessed for diagnostic criteria [1].

PCL patients often come to medical attention with symptoms of fatigue, tiredness, dyspnea on exertion (DOE), and bone pain. These often prompt work up revealing abnormal laboratory and imaging findings that initially suggest MM including anemia, bone pain, hypercalcemia, and renal failure [8]. However, patients with PCL often present with similar but worse features than MM. In one study of 80 PCL and 439 MM patients, PCL patients had significantly higher lactate dehydrogenase (LDH), creatinine, and WBC values compared to MM patients [9]. In fact, LDH and WBC are more frequently normal at presentation in MM patients; but when raised they often portend poor prognosis [10]. Additionally, they had 1.5g/dL of hemoglobin lower than MM patients. Interestingly, lytic lesions seem to occur more in MM patients than PCL patients [9]. Our patient had low hemoglobin with high WBC, LDH, and creatinine values at diagnosis.

What makes this case unique is that he presented with necrotic skin lesions in the upper and lower extremities concerning leucocytoclastic vasculitis; and with his anemia and renal failure, we thought of an autoimmune process with vasculitis, glomerulonephritis, and hemolytic anemia. However, work-up showed a negative autoimmune screen and high cryoglobulin (81%), and high blood viscosity (cp 3.1). Cryoglobulin is a very rare finding in PCL. We did not find any case reports or studies of cryoglobulinemia among PCL patients. There were very few case reports of MM and cryoglobulinemia [6,11].

Our patient had type I cryoglobulinemia, which though rare, occurs most commonly in patients with b-cell malignancies including Waldenstroms, MM, and chronic lymphocytic leukemia (CLL) [12]. The most common clinical feature in type I cryoglobulinemia is skin involvement including Raynaud’s, livedo reticularis, or necrotic lesions [11-13]. Other clinical features include arthralgias, renal failure, neuropathy, and central nervous system (CNS) involvement.

Cryoglobulinemia can be life-threatening because of the severity of skin, kidney, and CNS involvement. Initial management should involve assessing viscosity and doing plasmapheresis if indicated [14]. Our patient presented with severe lower extremity pain and necrotic lesions in the lower and upper extremities. Additionally, the patient had severe acute renal failure. His initial blood viscosity was 3.1 with 81% cryoglobulin. The patient underwent plasmapheresis and showed improvement in his creatinine level and cryoglobulin was undetected after few days.

Definitive treatment of cryoglobulinemia requires treating the underlying disorder. Our patient was started on treatment with Bortezomib, Lenalidomide, and Dexamethasone. Before the early 2000s, the mainstay of treatment involved chemotherapy with alkylating agents and vinca alkaloids. Overall response rates (ORR) were often less than 50% with overall survival (OS) ranging between four to seven months [4,5,15]. However, with the advent of novel agents (Proteasome inhibitors [PI] and immunomodulatory [IMiD]), survival among PCL patients has improved with additional survival benefits in patients who underwent ASCT. In one retrospective study of 50 PCL patients, patients on Bortezomib-based regimen (BBR) + ASCT had significantly longer progression-free survival (18 months vs. 9 months) and OS (48 months vs. 14 months) than patients on non-Bortezomib-based regimen (NBBR) [16]. In another retrospective study of 38 PCL patients, all the patients received a BBR and showed an ORR of 82%. The median PFS was 20 months and the median OS was 33 months [17]. While there seems a preponderance of retrospective studies on PCL, only two prospective studies have been published on PCL - a veritable testament to the rarity of this disease.

The first was a phase II trial among 24 patients who all received lenalidomide-dexamethasone (RD) with some eventually undergoing ASCT [18]. After a median follow-up of 34 months, median PFS was 14 months and median OS was 28 months. For those who underwent ASCT, the median PFS was 27 months while median OS was not reached. In the multivariable analysis, only ASCT and response to therapy impacted survival. Interestingly, high LDH and adverse cytogenetics had no impact on survival.

The second prospective study was done among 39 patients with PCL. All patients were initially treated with three to four cycles of a BBR, then those patients who responded, underwent ASCT [19]. Patients who underwent ASCT had longer OS (36.3 months vs. 10.5 months) compared to those who could not undergo ASCT. For our patient, at the time of manuscript writing, he was being prepared for ASCT.

## Conclusions

Our patient responded well to induction therapy with the bortezomib-based regimen. Most patients with PCL do not respond that well. PCL is a rare disease with a historically poor prognosis. Patients often present with clinical and laboratory features that are similar but worse than in multiple myeloma. We present a case of cryoglobulinemia in a PCL patient - a very rare complication in PCL patients. Timely recognition and adequate management of this complication can help prevent a fatality. Treatment of the underlying plasma cell leukemia should also follow. Though the prognosis of PCL has improved in the last decade, the outcomes are still poor compared to multiple myeloma. Thus the need for more research among patients with PCL.
